# The microbiome of a Pacific moon jellyfish *Aurelia coerulea*

**DOI:** 10.1371/journal.pone.0298002

**Published:** 2024-04-18

**Authors:** Aki H. Ohdera, Maille Mansbridge, Matthew Wang, Paulina Naydenkov, Bishoy Kamel, Lea Goentoro

**Affiliations:** 1 Division of Biology and Biological Engineering, California Institute of Technology, Pasadena, CA, United States of America; 2 National Museum of Natural History, Smithsonian Institute, Washington, D.C., United States of America; 3 Alverno Heights Academy, Sierra Madre, CA, United States of America; 4 Flintridge Preparatory School, La Cañada Flintridge, CA, United States of America; 5 US Department of Energy Joint Genome Institute, Lawrence Berkeley National Laboratory, Berkeley, CA, United States of America; Bristol-Myers Squibb Company, UNITED STATES

## Abstract

The impact of microbiome in animal physiology is well appreciated, but characterization of animal-microbe symbiosis in marine environments remains a growing need. This study characterizes the microbial communities associated with the moon jellyfish *Aurelia coerulea*, first isolated from the East Pacific Ocean and has since been utilized as an experimental system. We find that the microbiome of this Pacific *Aurelia* culture is dominated by two taxa, a Mollicutes and Rickettsiales. The microbiome is stable across life stages, although composition varies. Mining the host sequencing data, we assembled the bacterial metagenome-assembled genomes (MAGs). The bacterial MAGs are highly reduced, and predict a high metabolic dependence on the host. Analysis using multiple metrics suggest that both bacteria are likely new species. We therefore propose the names *Ca*. Mariplasma lunae (Mollicutes) and *Ca*. Marinirickettsia aquamalans (Rickettsiales). Finally, comparison with studies of *Aurelia* from other geographical populations suggests the association with *Ca*. Mariplasma lunae occurs in *Aurelia* from multiple geographical locations. The low-diversity microbiome of *Aurelia* provides a relatively simple system to study host-microbe interactions.

## Introduction

Jellyfish are increasingly recognized as an important player in ecosystem functions and biogeochemical cycles [1–4]. A recent estimate suggests that gelatinous zooplanktons facilitate transfer, annually, of 0.4–2.1 gigatons of carbon to the seafloor [5]––about 20% on average of the total 5–6 gigatons of carbon deposited annually to the seafloor [6]. Carbon deposition to the seafloor is an important mechanism for absorbing carbon from the world’s atmosphere, and buffering the effects of the increasing carbon dioxide emission from human activities [7]. The effects of jellyfish on the marine ecosystem and biogeochemical cycles are magnified during bloom events. During a bloom, the jellyfish population rapidly increases within a short period of time, in some instances by as much as 5000% [1]. The rapid increase in biomass impacts the food web dynamics [8] and alters carbon, phosphorus, and nitrogen cycles [9]. Nitrogen release by jellyfish during a bloom can be as high as supporting more than 100% of nitrogen required for daily primary production by phytoplankton [2, 10, 11]. Jellyfish blooms also have an economic impact, having disrupted the fishery and tourism industries on multiple occasions [12, 13]. There is evidence that jellyfish blooms may increase in frequency and amplitude in the future because of anthropogenic processes [14, 15], highlighting the need to better understand the biology of jellyfish.

Jellyfish from the genus *Aurelia* (Cnidaria: Scyphozoa) are highly abundant and widely distributed across the world [16]. *Aurelia* can thrive in wide-ranging environments, from tropical seas to subarctic regions, from open oceans to brackish estuaries [17, 18]. In addition to its prevalence, *Aurelia* is also increasingly utilized as a laboratory model for studying multiple biological processes [19], including metamorphosis [20], biomechanics [21], regeneration [22], and neuroscience [23]. As an early branching metazoan, *Aurelia* provides an evolutionary lens with which to probe the early forms of biological processes in animals. The genome of *Aurelia coerulea* was recently sequenced [24], further empowering the use of *Aurelia* as a comparative model. Finally, being one of the most energetically efficient propulsors on the planet [25], *Aurelia* is a biological model for engineering muscular pumps [26], robotic swimmers [26] and biohybrid ocean sensors [27].

In this study, we characterize the microbiome of *Aurelia coerulea*, originally collected from the Eastern Pacific Ocean, and has been cultured in the lab for ten years. Microbiome impacts many aspects of animal biology, including traits previously thought to be solely dependent on the host genotype, such as development and behavior [28]. Indeed, in *Aurelia* polyps, modulation of microbiome impacts asexual reproduction, feeding rate, and growth [29]. There have been several studies sequencing the microbiome of *Aurelia* from different geographical locations [30–36]. These studies revealed that although the microbial communities of *Aurelia* are regionally different, there are interesting overlaps in the bacterial taxa, which motivate the need for more comparative studies of microbiomes from different geographical populations. In this study, we analyzed the microbial communities of *Aurelia* originating from the Pacific population, the origin of the strain whose genome has recently been sequenced [24]. Our study contributes to the existing literature by expanding the comparative analysis of *Aurelia* across geographical populations as well as characterizing an *Aurelia* strain that is increasingly being used as a laboratory model.

## Materials and methods

### Jellyfish culture

Polyp cultures were established from polyps originally collected by the Cabrillo Marine Aquarium (San Pedro, CA) from Long Beach (33°46′04.2′′N 118°07′44.2′′W, GPS: 33.7678376–118.1289559). We refer to this population of *Aurelia coerulea* as *Aurelia* AcGM. Polyps were maintained in artificial sea water (ASW) at 22°C on a 12:12 light-dark cycle and fed 48 hr old *Artemia* brine shrimp enriched with RGComplete (Reed Mariculture, USA) every two days. Polyps were induced to strobilate using 5-methoxy-2-methyl-indole (Sigma-Aldrich, USA) at 25 mM in sea water [37]. Polyps were exposed to the inducer overnight, and rinsed three times with ASW prior to transfer to 1 liter Imhoff cones (Nalgene, USA) with aeration. Induced animals were fed with *Artemia* every 48 hrs leading up to strobilation. To obtain the medusa stages, ephyrae were grown in an Imhoff cone and fed rotifers every day until animals were approximately 1 cm in diameter.

#### Amplicon sequencing

Individual animals were collected and rinsed three times with 0.22 μM sterilized artificial sea water. To minimize contamination from their diet, animals were starved for 24 hrs prior to sampling. For ephyrae, animals that were less than 4 days old post-strobilation were collected. For medusae, 1 cm diameter animals were collected. Excess water was removed by blotting animals on parafilm using forceps. For each sample, 3–5 individuals were pooled. For each stage (polyp, ephyra, and medusa), 5–6 total samples were prepped. Animals were flash frozen in liquid nitrogen and stored at -80°C until further processing. Four sea water controls were prepared by filtering 500 ml of ASW through a 4.7 mm diameter 0.45 μM cellulose nitrate membrane filter (Whatman, United Kingdom). Half of the membrane was cut from the disc and used for the DNA extraction. DNA from flash frozen animals and controls were extracted using the Qiagen DNeasy Powersoil kit (Qiagen, Germany) following the manufacturer’s protocol. Four kit controls were prepared by performing the extraction procedure with a set of blank extraction columns. Amplification of the V4 region of the 16S rRNA gene was performed using a modified 515F–806R primer set [38]. We chose to target the V4 region because it has been shown to be effective in studies of cnidarian-associated microbiomes [39, 40]. We find the modified primer sets performed better than the standard 515F-806R set in reducing amplification of host mitochondrial DNA. PCR amplification was performed with the NEB Q5 High-fidelity master mix (New England Biolabs, USA). Amplification was performed in triplicate for each sample. Replicate reactions were combined and amplification was confirmed on a 1% agarose gel. Samples were purified using the Qiagen QIAquick PCR Purification kit (Qiagen, Germany). Purified amplicons concentrations were quantified using the Qubit dsDNA HS Assay kit (cat. Q32851). Purified samples were submitted to Genewiz or the Georgia Genomics and Bioinformatics Core for 300-bp and 250-bp paired-end sequencing, respectively, on the Illumina MiSeq. 16S rRNA gene amplicon sequencing data have been submitted to NCBI under BioProject PRJNA975886.

#### Amplicon sequencing analysis

Quality of sequenced reads were checked with FastQC (ver. 0.11.9; https://www.bioinformatics.babraham.ac.uk/projects/fastqc/). Reads were analyzed with the DADA2 function of Qiime2 (ver. 2022.2) [41]. Reads were trimmed for quality and chimeric reads were removed with the *denoise-paired* function of dada2 with the following parameters:—p-trunc-len-f 177—p-trunc-len-r 170—p-trim-left-f 5—p-trim-left-r 10. Trimming parameters were selected using Figaro [42]. Taxonomic assignment of denoised, paired reads was performed using the Qiime2-formatted classifier (http://docs.qiime2.org/2023.9/data-resources/) pre-trained with V4 region sequences (515–806 bp) extracted from the Silva 16S rRNA database (version 138_99) [43]. Reads were then filtered using the *filter-samples* and the *filter-features* function of qiime2 to remove low-count sequences and singletons. Only samples with a minimum of 3500 reads were retained. Of the remaining samples, features were minimally required to be present in 2 samples and totaling 100 reads. Features that did not meet these requirements were removed prior to downstream analysis. Rarefaction curves were generated with the *diversity alpha-rarefaction* function of Qiime2 to ensure adequate coverage and depth of sequencing. 12,000 reads were sub-sampled, which is approximate to the lowest read depth of all jellyfish samples (S1 Fig in S9 File). To further determine whether we have identified genuine microbiome members of *Aurelia* AcGM, we used the R package decontam [44]. Decontam compares control and real samples and employs statistical methods to identify likely DNA contamination. Phylogenetic analysis of the retained features was performed with the *align-to-tree-mafft-fasttree* function of qiime2 using standard settings.

#### Assembly of bacterial MAGs from host genome sequencing data

Sequences generated for the assembly of the *A*. *coerulea* genome were retrieved from the National Center for Biotechnology Information (NCBI) [45] (Accession PRJNA490123). Initially, barcodes were removed from the reads and quality filtered using Trimmomatic-0.39 with standard parameters [46]. The trimmed reads were assembled with MegaHit (ver. 1.2.9), which incorporates DNA composition and abundance of unique sequences of DNA (k-mers) to group reads during the assembly process [47]. The assembly was performed with the following settings:—k-min 25—k-max 115—k-step 10—min-contig-len 300 -m 0.4. Assembled contigs were binned into MAGs using MetaBAT (version 2.12.1) [48], Vamb (ver. 4.1.3) [49], and Rosella (ver. 0.4.2) (https://rhysnewell.github.io/rosella/). We chose to include Rosella because it incorporates long read information into the binning process. Consensus bins were generated using DAS Tool (ver. 1.1.6) (S1 Table in S10 File) [50]. We performed a BLAST search of the five longest sequences against the NCBI nt database to determine whether the binned MAGs were comprised of host or bacterial sequences. Binned contigs were then scaffolded using sspace-standard (ver. 3) or sspace-longread [51, 52]. Paired-end read libraries (SRR7889280, SRR7866920) and mate-paired libraries (4000 bp; SRR7834587, 8000 bp; SRR7866321) were used as input for sspace-standard. Pacbio reads (SRR7866923) were used as input for sspace-longread. Resulting scaffolded genomes with greater length and fewer contigs were chosen for subsequent processing and analysis. Gaps were closed with LR_Gapcloser [53]. Polishing of the scaffolded MAGs was performed using Pilon [54]. Briefly, paired-end libraries were aligned to the scaffolded genomes using BWA (ver. 0.7.12-r1039) [55] to generate bam format files and sorted using Samtools (ver. 1.15.1) [56]. Host sequences were identified from the MAGs using BLASTx against the nr database. Gene prediction was performed with the Rapid Annotation using Subsystem Technology tool kit (RASTtk) pipeline [57, 58]. The MAGs have been submitted to NCBI under BioProject PRJNA975886.

Quality of the MAGs was assessed using multiple metrics. Genome completeness and contamination was quantified using CheckM, as part of the Protologger galaxy web application (ver. 0.99) [59, 60]. A homology based method employed in the MiGA Online web server was additionally used to confirm the CheckM results [61]. CheckM performs the quality assessment using a taxonomically relevant set of marker genes. MiGA utilizes a set of essential genes defined in Dupont et al. [62] for a homology search performed by HMMER [63]. Taxonomic identification of the binned contigs was performed by comparing the 16S rRNA gene and the whole genome to existing databases. Full length 16S rRNA gene sequences were retrieved using Bedtools (ver. 2.26.0) [64] for *Aurelia* Mollicutes and *Aurelia* Rickettsiales to perform a homology search against the NCBI rRNA/ITS database with BLASTn, with uncultured/environmental sample sequences excluded [65, 66]. Relatedness to existing bacterial genomes was computed with average nucleotide identity (ANI) values using GTDB-Tk [67] as part of the Protologger pipeline [59], MiGA Online [61], as well as using the Type Strain Genome Server (TYGS) [68]. In addition to the Mollicutes and Rickettsiales MAGs, a third bacterial bin of roughly 4 Mb was recovered. However, the third bin is of low quality, possibly coming from lower-abundance symbionts. Further analysis of this third MAG therefore needs more directed sequencing.

#### Comparative analysis of the assembled MAGs

To assess the degree of genome reduction, we compared protein coding sequence content and genome length of the *Aurelia* Mollicutes MAG and *Aurelia* Rickettsiales MAG with those of existing genomes. Representative genomes, defined by the NBCI according to a set of predefined criteria (www.ncbi.nlm.nih.gov/refseq/about/prokaryotes/#representativegenome) were retrieved from NCBI RefSeq. A total of 128 representative genomes were retrieved for the Mollicutes (See S7 File for RefSeq accessions). An additional genome of *Ca*. Spiroplasma holothuricola was included (GCA_002135175.2) [69] in the analysis. A total of 82 representative Rickettsiales genomes were retrieved from NCBI (See S8 File for RefSeq accession). *Ca*. Aquarickettsia rohweri was also included in the analysis (GCF_003953955.1) [70]. Coding sequence and genome size were plotted with ggplot2 (ver. 3.4.0) in R (ver. 4.1.0).

#### Metabolic analysis with NetCooperate

The degree of metabolic reliance by the assembled genomes on the jellyfish host was quantified using NetCooperate [71]. Genome-scale metabolic modeling of *A*. *coerulea* as well as the *Aurelia* Mollicutes and *Aurelia* Rickettsiales MAGs was performed with PRIAM using the predicted coding sequences as input [72]. The resulting sbml file was converted to a network format with a custom perl script prior to the analysis with NetCooperate. Draft metabolic models generated from the MAGs for each bacterial taxon were paired with a draft metabolic reconstruction of the *Aurelia* genome to calculate the biosynthetic support scores (BSS). Enzymatic reaction equations were parsed for its constituent metabolites, with products and substrates connected by edges. Completeness of metabolic pathways were manually confirmed against the KEGG database using KofamKoala [73]. Genomic data for *Aurelia* associated bacteria and the script used for NetCooperate data processing have been uploaded to the open repository CaltechDATA (https://doi.org/10.22002/371wp-5nb51).

#### Phylogenetic tree construction

Reconstruction of the *Aurelia* Mollicutes and *Aurelia* Rickettsiales phylogeny was performed using both a concatenated set of single-copy orthologs and the ribosomal 16S rRNA gene. Single-copy orthologs were identified using OrthoFinder (ver. 2.5.4) [74]. A subset of genomes for each taxa were used as input for the orthogroup analysis due to computational constraints. Identified single-copy orthologs were retrieved from the protein predictions and manually confirmed to be single copy for all genomes included in the analysis. A total of 11 single-copy orthologs were identified for the Mollicutes and 56 single-copy orthologs for Rickettsiales. Mafft (ver. 7.4.29) [75] was used for alignment of both single-copy ortholog sequences and 16S rRNA gene sequences with the accuracy-oriented method (L-INS-i) with 1000 cycles of iterative refinement. Aligned sequences were trimmed with BMGE (ver. 1.12) [76] with default parameters. For the single-copy ortholog tree, aligned sequences were concatenated using catsequences (ver. 1.4) (https://zenodo.org/record/7956648). Maximum-likelihood phylogenetic reconstruction with ultrafast bootstrapping was performed with IQ-TREE (ver. 2.03) [77, 78] with the following parameters: -alrt 1000 -bb 2000 -bnni -m MFP. Model selection and partition finding was performed with ModelFinder [79] and PartitionFinder. The resulting tree was visualized and modified in iTOL [80].

## Results

### The microbiome of *Aurelia coerulea* AcGM is dominated by two taxa

**Fig 1:**
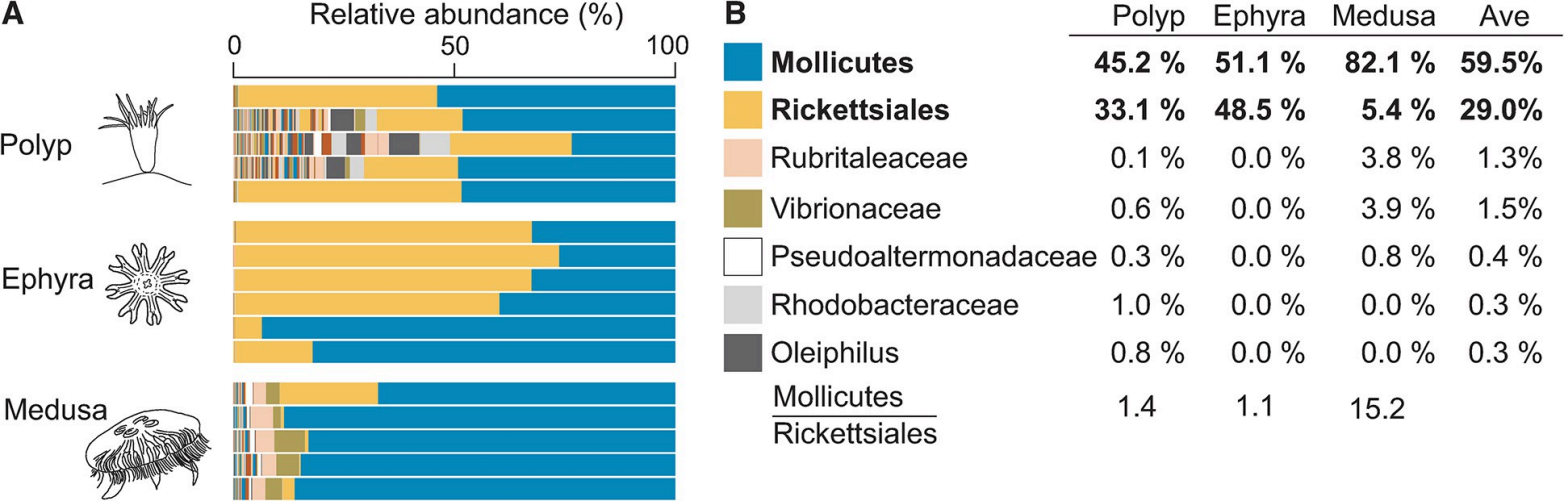
The bacterial microbiome of *Aurelia* AcGM is dominated by two taxa. **(A)** To characterize the jellyfish-associated microbiome associated with *Aurelia* ACGM, we performed next-generation amplicon sequencing of the V4 region of the 16S rRNA gene. Each biological replicate comes from 3–5 animals. The top ten taxa, color-coded, are listed in Fig 1B. **(B)** The top ten most abundant taxa present in *Aurelia* AcGM across life stages. Percent abundance shown is the average across biological replicates. The last column shows average (‘Ave’) across life stages. In the last row, the ratios of Mollicutes and Rickettsiales abundance are computed across life stages.

Our lab population of *Aurelia coerulea*, which we refer to as *Aurelia* AcGM, was originally collected from the East Pacific Ocean (the GPS coordinates given in Methods). It was classified as *Aurelia aurita* sp. 1, and recently revised to *Aurelia coerulea* [81]. To determine the bacterial composition of the *Aurelia* microbiome, we performed 16S amplicon sequencing (Fig 1A; S2 Table in S10 File provides the complete sequencing results). We recovered a total of 197 amplicon sequence variants, 95% of which could be identified to at least the phylum level and 78% of which to the genus level. To verify that the bacterial sequences represent animal-associated microbes and not a carryover from the environmental microbiome, we sequenced and verified that the ambient artificial sea water and the kit reagents contain distinct bacterial compositions (S2 Fig in S9 File, S3 Table in S10 File), confirming that the bacterial sequences obtained from *Aurelia* are genuine members of the *Aurelia* microbiome.

We grouped the bacterial amplicon sequences by their lowest taxonomic classification (Fig 1A). We found that the *Aurelia* AcGM microbiome is dominated by a bacterium from the class Mollicutes (phylum Firmicutes, recently renamed as Mycoplasmatota) and a bacterium from the order Rickettsiales (phylum Proteobacteria, also called Pseudomonadota). Mollicutes and Rickettsiales make up on average 89% of the amplicon reads across the life stages (Fig 1B). The next five most abundant taxa each comprises 1.5% or less of the total bacterial abundance (Fig 1B). We conclude that the microbiome of *Aurelia* AcGM in our population is dominated by Mollicutes and Rickettsiales.

The relative composition of Mollicutes and Rickettsiales varies across life stages. Like other jellyfish, *Aurelia*’s life cycle consists of two post-larval forms, the sessile polyps and free-swimming medusae (Fig 1A). In *Aurelia* and many scyphozoans, the transition from polyps to medusae involves an ephyra stage, a hardy stage that facilitates dispersal. In *Aurelia* AcGM polyps and ephyrae, Mollicutes and Rickettsiales are found at approximately equal ratios (1.4:1 in polyp and 1:1 in ephyra; Fig 1A and 1B). Strikingly, the medusa stage is characterized by a dramatic increase in Mollicutes, at 15-fold greater abundance than Rickettsiales (Fig 1A and 1B).

### Association with Mollicutes recurs in several geographical populations

**Fig 2:**
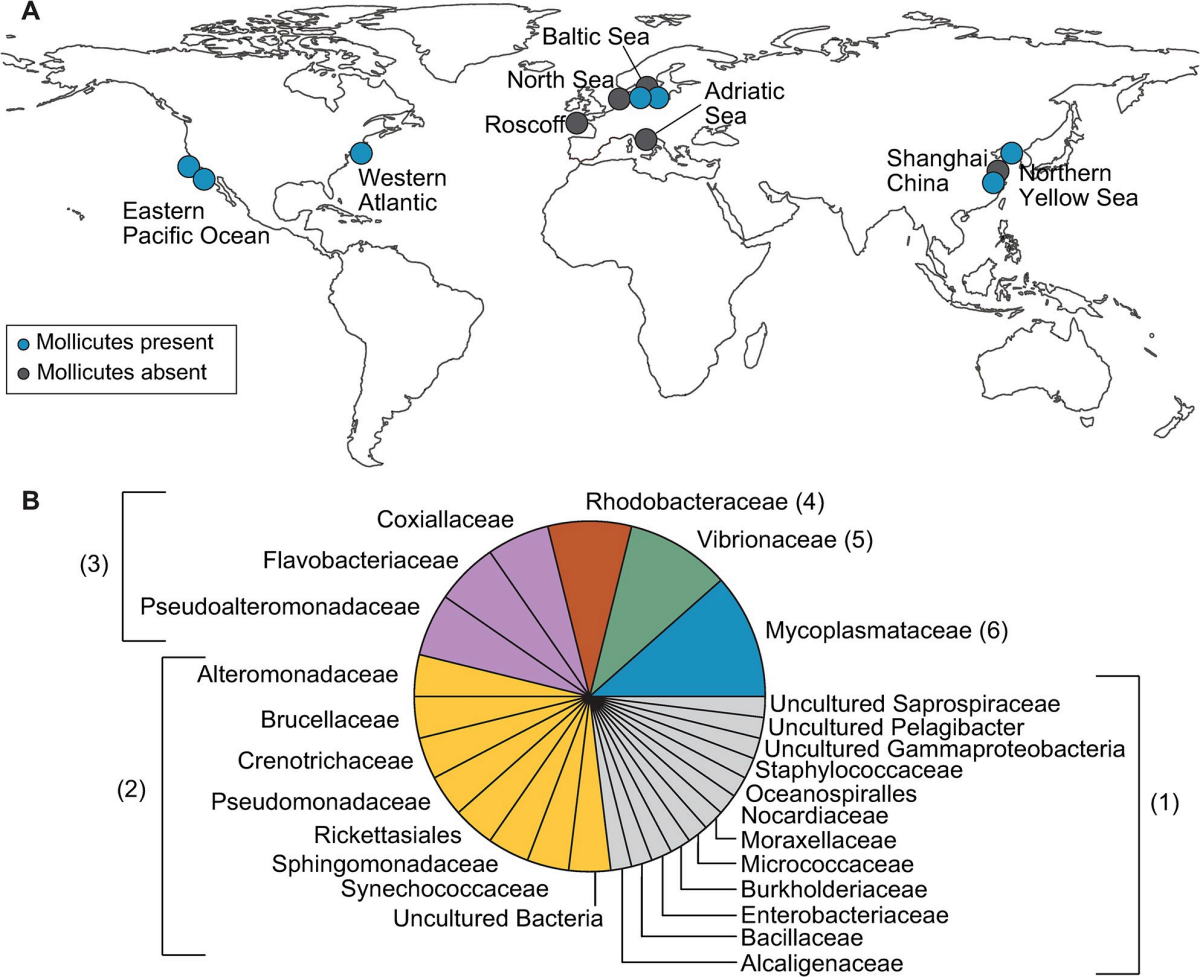
Microbiome composition of *Aurelia* varies across geographical populations. **(A)** Geographic locations of *Aurelia* populations that have been analyzed so far for their microbiome composition. **(B)** Bacterial families found to associate with *Aurelia* studied so far. Families included in this pie chart make up at least 5% of the relative abundance. We plotted the family level because this is the lowest common taxonomic classification reported in all of the studies. The numbers in parentheses indicate the number of geographical locations in which the families were found to associate with an *Aurelia* species. The colors simply group the families based on the number of geographical occurrences. See S4 Table in S10 File for the detailed survey of the existing *Aurelia* microbiome studies.

Next, we surveyed existing reports to assess how the microbiome of *Aurelia* AcGM compares to those of other *Aurelia* populations. Microbiome composition varies across *Aurelia* from different parts of the world (S4 Table in S10 File lists the taxa found across *Aurelia* populations) [30–36]. However, we notice recurring patterns. First, across the geographical populations analyzed so far, the *Aurelia* microbiome tends to be of a low-diversity, consisting of 2–5 dominant taxa. There are no clear differences between microbiome characterized from lab vs wild specimens, i.e., microbiome of lab specimens is not necessarily less complex or show distinct bacterial communities. Second, several bacterial families recur, as summarized in Fig 2B. Highest in frequency, although ranging in abundance, is an association with a Mollicutes, found in six out of the twelve populations analyzed so far (Table 1). Third, most of the Mollicutes, remarkably, appear to be closely related (S4 Table in S10 File). *Aurelia* from the Atlantic, Baltic, China, and Pacific, each associate with a Mollicutes that shows >97% sequence similarity to the Mollicutes found in *Aurelia* AcGM, as analyzed by BLAST analysis of the 16S amplicon sequences, suggesting that the Mollicutes found in these different geographical populations are likely the same species.

**Table 1:** *Aurelia* from several parts of the world associate with a Mollicutes likely of the same species. We surveyed existing studies that characterize bacteria that associate with *Aurelia* for Mollicutes association. S4 Table in S10 File provides the detailed taxa composition recovered from each study.

	Geographical origin	Specimen	Mollicutes abundance	Life stage analyzed	References	Similarity to AcGM Mollicutes
1	Atlantic Ocean, US	Wild	20%	Medusa	30	97%
2	Pacific Ocean, US	Lab culture	45–82%	Polyp, ephyra, medusa	This study	100%
3	Pacific Ocean, US	Lab culture (San Diego strain)	Abundant in host sequencing data	Polyp, ephyra, medusa	This study; mining data from ref. 24	100%
4	Roscoff, France	Lab culture	Absent	Polyp, strobila, ephyra, medusa	31	N/A
5	Baltic Sea	Lab culture	Absent	Polyp	31	N/A
6	Baltic Sea	Wild	42–90%	Medusa	31	75%
7	Baltic Sea	Wild	9%	Medusa	32	98%
8	North Sea	Lab culture	Absent	Polyp	31	N/A
9	Adriatic Sea	Wild	Absent	Medusa	33	N/A
10	Shanghai, China	Aquafarm	Absent	Medusa	34	N/A
11	Shanghai, China	Aquafarm	9–17%	Medusa	35	97%
12	Northern Yellow Sea,	Wild	1%	Medusa	36	97%

### Metagenome-assembled genomes of *Aurelia* AcGM-associated microbes

**Fig 3:**
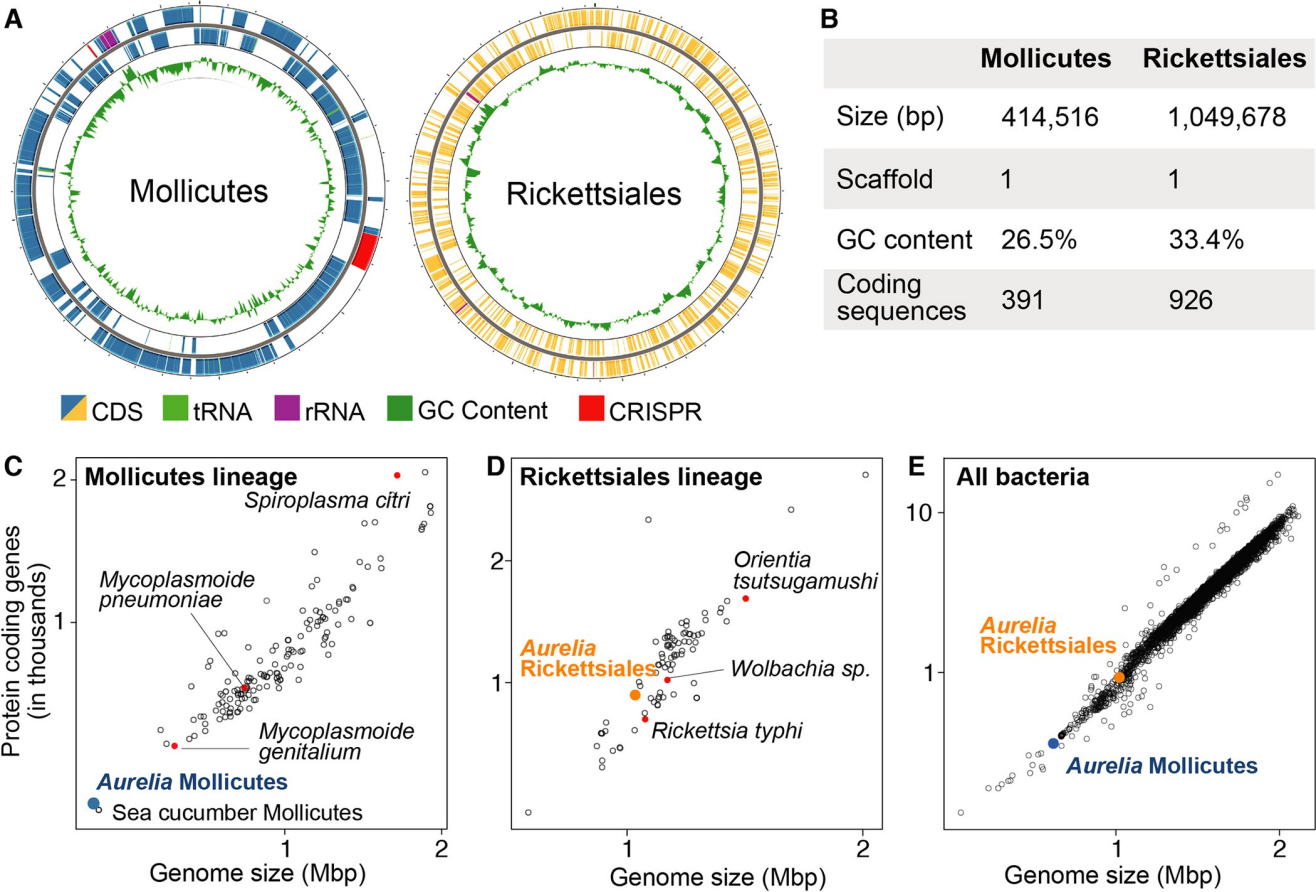
Metagenome-assembled genomes (MAGs) of the *Aurelia* AcGM-associated Mollicutes and Rickettsiales. The MAGs were recovered from the host genomic sequencing reads [24]. **(A)** Circos plots of *Aurelia* Mollicutes and *Aurelia* Rickettsiales MAGs. **(B)** Assembly statistics for the *Aurelia* Mollicutes and *Aurelia* Rickettsiales MAGs. More detailed statistics are described in S5 Table in S10 File. **(C-E)** Representative genomes of bacterial species were retrieved from the NCBI RefSeq database. Genome size and number of protein coding sequences were plotted for Mollicutes **(C)**, Rickettsiales **(D)** and representative genomes of all accepted bacterial species **(E)**. Genome size and number of protein coding sequences are log10 transformed for visualization.

To characterize the *Aurelia* Mollicutes and Rickettsiales, we mined the publicly available host genome sequencing data [24] to assemble draft bacterial genomes. For Rickettsiales, we recovered a 1 Mb uncircularized metagenome-assembled genome (MAG; Fig 3A and 3B). The Rickettsiales MAG was 95.5% complete with 0.2% contamination. The Rickettsiales MAG contains 91.5% of essential bacterial genes. Finally, the assembled Rickettsiales MAG is within the expected size for the Rickettsiales group (Fig 3D), containing 926 protein coding genes. The genome contains 38 tRNA cognates for all 20 essential amino acids. These metrics suggest that the draft Rickettsiales MAG, although uncircularized, provides a good coverage.

**Fig 4:**
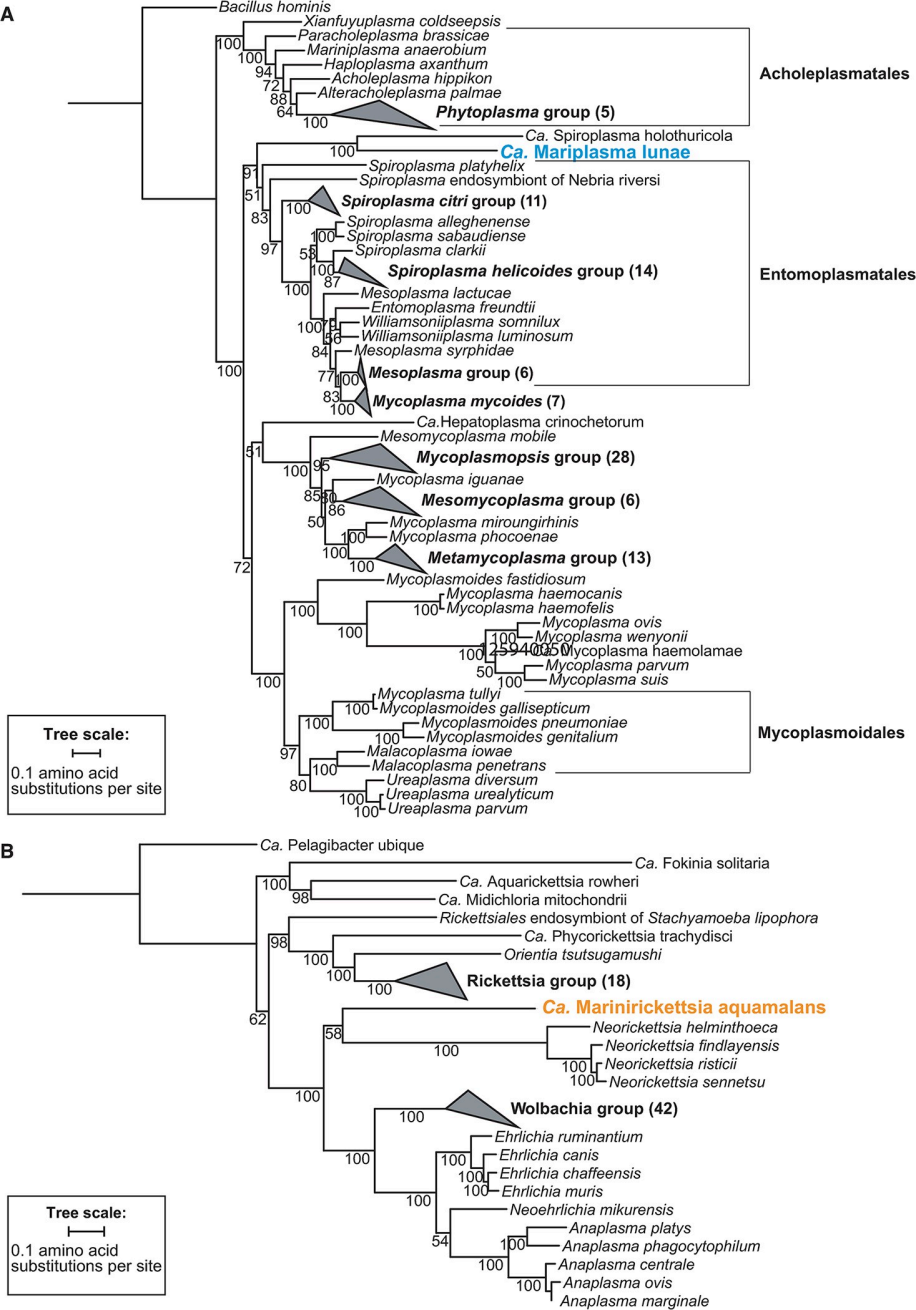
Bacteria that associate with *Aurelia* AcGM are likely new species. A concatenated maximum-likelihood phylogenetic tree was constructed using IQ-TREE with ultra-fast bootstrapping (n = 2000). Ultrafast bootstrap support values are shown for each branch point. The number of species comprising collapsed branches are shown in parentheses. **(A)** Phylogenetic reconstruction of the *Aurelia* Mollicutes. Eleven single-copy orthologs from 128 Mollicutes species were analyzed. **(B)** Phylogenetic reconstruction of the *Aurelia* Rickettsiales. Fifty-six single-copy orthologs from 82 Rickettsiales species were analyzed.

For Mollicutes, we successfully assembled a complete, circularized genome of 0.4 Mb in size (Fig 3A and 3B), with a 2.6% contamination. The Mollicutes MAG was assembled with a read depth of 2800X, giving us confidence that we have recovered the full genome. Mollicutes are known for having small genome sizes, having undergone genome reduction. The *Aurelia* Mollicutes MAG consists of 391 protein coding genes—and is one of the smallest genomes described to date (Fig 3C and 3E). Gene completeness analysis shows that the *Aurelia* Mollicutes MAG contains only 80% of known Mollicutes marker genes and 72% of essential bacterial genes, suggesting that it is a reduced genome. Of the representative Mollicutes genomes available in the NCBI database (Fig 3C), the most closely related to *Aurelia* Mollicutes is the one associated with sea cucumber, *Ca*. Spiroplasma holothuricola (the phylogenetic relationship will be analyzed in Fig 4), which has a similar genome size to the *Aurelia* Mollicutes (Fig 3C, [69]).

### *Aurelia* AcGM-associated microbes rely on host for nutrients

The small genomes suggest that Rickettsiales and Mollicutes rely on the *Aurelia* host for nutrients. To assess the degree of metabolic coupling, we performed a pairwise NetCooperate analysis between the draft metabolic reconstructions of each bacterium and the host *Aurelia* [71]. NetCooperate calculates a Biosynthetic Support Scores (BSS), which reflects the degree to which genome 1 relies on genome 2 for metabolites. A BSS score of 1 reflects a complete reliance. The BSS score of *Aurelia* Rickettsiales is 0.84, and the BSS score of *Aurelia* Mollicutes is 0.91—suggesting a high degree of metabolic reliance on the host.

Indeed, directly assessing the genomes for absence/presence of metabolic pathways suggest reduced metabolic capacities (S6 and S7 Tables in S10 File). *Aurelia* Rickettsiales has a complete TCA pathway, but lacks the glycolysis pathway that supports the input to the TCA pathway, as well as most of the pentose phosphate pathway (PPP). The lack of glycolysis appears to be prevalent in Rickettsiales, as analysis of Rickettsiales genomes from the NCBI database confirms widespread loss of glycolytic enzymes (S6 Table in S10 File). Lacking glycolysis to generate pyruvate, Rickettsiales likely employ alternative methods to fuel the TCA and anabolic pathways. Pyruvate can be synthesized from intermediates of the PPP, intermediates of Entner-Doudoroff pathway, or from lactate and alanine. However, *Aurelia* Rickettsiales does not have the necessary enzymes to do so. Therefore, it is likely that *Aurelia* Rickettsiales uptake pyruvate from the host. Finally, *Aurelia* Rickettsiales are missing about half of the amino acid biosynthesis pathways, and therefore likely acquire multiple amino acids from the host, as evidenced by amino acid transporters in the genome (S7 Table in S10 File). Other species of Rickettsiales are also known to uptake amino acids [82–84]. Amino acids can also fuel the TCA cycle. *Aurelia* Rickettsiales has the genes that facilitate conversion of glutamine to glutamate and ultimately to alpha-ketoglutarate, a TCA intermediate. *Aurelia* Rickettsiales also has the enzymes for converting proline to glutamate and aspartate to oxaloacetate, another TCA intermediate. Finally, Rickettsiales are known to acquire vitamins and cofactors from their host [85]. Indeed, *Aurelia* Rickettsiales appears incapable of synthesizing vitamins, therefore likely acquiring them from the host. *Aurelia* Rickettsiales is capable of synthesizing heme A, but likely acquire other cofactors from the host.

Compared to Rickettsiales, the even smaller Mollicutes genome suggests more reliance on host (S7 Table in S10 File). Key energy-generation pathways—glycolysis, TCA, and pentose phosphate pathways—are all missing from the Mollicutes genome. As an alternative energy-generating pathway, the Mollicutes genome encodes the arginine deiminase (ADI) pathway, which generates ATP by metabolizing arginine. The ADI pathway has indeed been utilized in other Mollicutes species [86]. As Mollicutes lacks the ability to synthesize most essential amino acids, Mollicutes likely acquire amino acids from the host. We verify the presence of an ABC transporter that can transport multiple metabolites, as well as the arginine-ornithine antiporter required for the ADI pathway (S7 Table in S10 File). Despite the uptake requirements of the *Aurelia* Mollicutes genome, only two genes were annotated as transporters. This suggests *Aurelia* Mollicutes transporters are capable of up-taking a broad set of metabolites, or unannotated genes have unidentified transporter functions. Finally, genes responsible for vitamin and cofactor biosynthesis are completely absent from the *Aurelia* Mollicutes genome, suggesting the bacterium is totally reliant on the host for these molecules.

### *Aurelia* Mollicutes and Rickettsiales are likely new species

Using the bacterial genomes, we performed taxonomic classification with the Genome Taxonomy Database toolkit (GTDB-Tk; [67]). Taxonomic classification is assigned by calculating a relative evolutionary divergence (RED) value and the average nucleotide identity relative to reference genomes in the GTDB database [67]. We failed to determine an average nucleotide identity (ANI) values with high confidence for both genomes (S5 Table in S10 File), due to their dissimilarities to known genomes within the respective lineages. The GTDB-Tk analysis suggests the *Aurelia* Mollicutes and Rickettsiales likely belong to novel genera of bacteria: Mollicutes potentially in the family Spiroplasmataceae, and Rickettsiales in the family Anaplasmataceae. To further corroborate this finding, we performed a digital DNA-DNA hybridization analysis (dDDH; S5 Table in S10 File). The dDDH analysis fails to find any genome similar to the Mollicutes or Rickettsiales MAG with a score higher than 30%, verifying that the two bacteria associated with *Aurelia* AcGM are likely novel taxa.

We next performed homology analysis using the 16S rRNA genes, whose full sequences were recovered from the Mollicutes and Rickettsiales MAGs. We find that the Mollicutes 16S rRNA gene is most similar to *Spiroplasma platyhelix*, with an 80% sequence similarity (S1 File). The Rickettsiales 16S rRNA gene is most similar to *Ehrlichia chaffeensis*, with a sequence similarity of 85.2% (S2 File). Species-level matches typically fall in the range of >97% similarity. The homology analysis to find good homologies for the 16S rRNA genes further supports the findings from the average nucleotide identity and digital DNA-DNA hybridization analyses that the *Aurelia* AcGM-associated microbes are likely new species.

In order to better resolve the phylogenetic position of *Aurelia* associated bacteria to known Mollicutes and Rickettsiales, we built phylogenetic trees using single-copy orthologs (Fig 4, S3 File). The *Aurelia* Mollicutes is most closely related (sister) to another Mollicutes associated with a marine invertebrate, *Ca*. Spiroplasma holothuricola, a Mollicutes associated with sea cucumbers. It is also interesting that both Mollicutes also have similar genome sizes (both on the small end; Fig 2). Although the two species group together relative to all other Mollicutes, they themselves appear to have undergone some divergence, as indicated by the long branch lengths from the split. Altogether, these results support the placement of *Aurelia* Mollicutes as a unique genus within the Mollicutes, sister to the Entomoplasmatales. To further verify the single-copy ortholog tree, we performed phylogenetic reconstruction with the 16S rRNA gene (S3 Fig in S9 File, S4 File), and obtained a similar taxonomic placement of *Aurelia* Mollicutes. We therefore propose the name *Ca*. Mariplasma lunae, to signify a new marine genus related to Spiroplasma that dwells in moon jellyfish.

Phylogenetic reconstruction of the Rickettsiales order places the *Aurelia* Rickettsiales with high confidence as a sister clade to the Wolbachia/Anaplasma/Ehrlichia group (Fig 4B, S5 File). The support score at the branch point between *Aurelia* Rickettsiales and *Neorickettsia*, the position of the *Aurelia* AcGM-associated bacteria is unclear. However, consistent with the single-copy ortholog analysis, analysis with the 16S rRNA gene consistently places the *Aurelia* Rickettsiales as sister to *Neorickettsia*, and with greater support (S4 Fig in S9 File, S6 File). Despite the 16S rRNA gene tree, greater taxonomic sampling is necessary within the *Aurelia* Rickettsiales-*Neorickettsia* clade to fully resolve the relationship. Regardless of the precise placement, both phylogenetic inference and genome similarity metrics strongly support the classification of the *Aurelia* Rickettsiales as a new genus. We therefore propose the name *Ca*. Marinirickettsia aquamalans for a new genus and species of Rickettsiales that associates with jellyfish.

## Discussion

In this study, we characterized the bacterial microbiome of the moon jellyfish *Aurelia coerulea*, originally collected from the Eastern Pacific Ocean and has been cultured in the lab for ten years, which we call *Aurelia coerulea* AcGM. We find that *Aurelia coerulea* AcGM has a low-diversity bacterial microbiome, dominated by two taxa. A low-diversity microbiome appears to be a stable feature of *Aurelia*, as the microbiome of *Aurelia* from other geographical locations, lab and wild specimens, consistently reveal 2–5 dominant taxa [30–36].

The Pacific *Aurelia* AcGM that we analyzed associates with bacteria from two taxa, a Mollicutes and a Rickettsiales. The diversity of the *Aurelia* microbiome is stable throughout its life stages, but the relative abundance of the Mollicutes and Rickettsiales changes. In particular, Mollicutes are significantly enriched in the medusa stage. Analysis using multiple metrics (average nucleotide identity, 16S rRNA homology, multi-gene phylogenetic analysis, and 16S rRNA taxonomy) suggests that *Aurelia* Mollicutes, as well as *Aurelia* Rickettsiales, are likely new genera. We therefore propose the names *Ca*. Mariplasma lunae (Mollicutes) and *Ca*. Marinirickettsia aquamalans (Rickettsiales).

Association with a Mollicutes appears to extend beyond our Pacific strains, as it has also been found in *Aurelia* from multiple other geographical populations, both in lab or wild specimens [30, 31, 34]. Remarkably, the 16S amplicon sequences of the *Aurelia*-associated Mollicutes from these previous studies show 97–100% sequence similarity to the Mollicutes we identify in *Aurelia* AcGM (Table 1 and S4 Table in S10 File). This suggests that association of *Aurelia* and *Ca*. Mariplasma lunae may be convergent across biogeographies and environmental conditions, potentially pointing to the existence of an *Aurelia* Mollicutes. On the other hand, association with Rickettsiales has been observed in only one other study [30], suggesting a more opportunistic relationship.

Mollicutes are known for having small genome sizes [86]. *Mycoplasmoides genitalium* with 0.58 Mb genome and 470 protein coding sequences [87] is among the smallest free-living forms of life, and a model system in minimal genome research. The *Aurelia* Mollicutes has 0.4 Mb and almost 100 fewer coding sequences than *M*. *genitalium*. Together with the sea cucumber associated Mollicutes, these are the smallest Mollicutes identified so far (Fig 3C) and among the smallest known bacterial genomes known so far (Fig 3E). Marine Mollicutes are much less studied than their land counterparts. Some of the most well-known Mollicutes are parasites studied for their impact on crop plants (e.g., *Spiroplasma citri*) and human health (e.g., *Mycoplasma pneumoniae*, *Mycoplasma genitalium*). The finding of a new marine species of Mollicutes in an animal host that can be studied in the lab provides an opportunity to add to our understanding of marine Mollicutes biology. At the same time, *Aurelia* is one of the most widespread jellyfish with increasing ecological impact due to their population dynamics and resilience in wide-ranging environments. It will be interesting to understand next how association with Mollicutes impacts *Aurelia* biology, and why association with Mollicutes appears to be recurrent across geographical populations of *Aurelia*.

## Supporting information

S1 File (XLSX)

S2 File (XLSX)

S3 File (TXT)

S4 File (TXT)

S5 File (TXT)

S6 File (TXT)

S7 File (CSV)

S8 File (CSV)

S9 File (DOCX)

S10 File (XLSX)
