# Supplementary material for: The microbiome of a Pacific moon jellyfish Aurelia coerulea
Source: PLoS One. 2024 Apr 18;19(4):e0298002. doi: 10.1371/journal.pone.0298002 (PMC11025843; doi:10.1371/journal.pone.0298002)
Supplement: S9 File — (DOCX) [file pone.0298002.s009.docx]

Supplementary Figures

**The microbiome of a Pacific moon jellyfish *Aurelia coerulea***

Aki Ohdera, Maille Mainsbridge, Matthew Wang, Paulina Naydenkov, Bishoy Kamel, Lea Goentoro

# Content

Supplement Figure 1

Supplement Figure 2

Supplement Figure 3

Supplement Figure 4


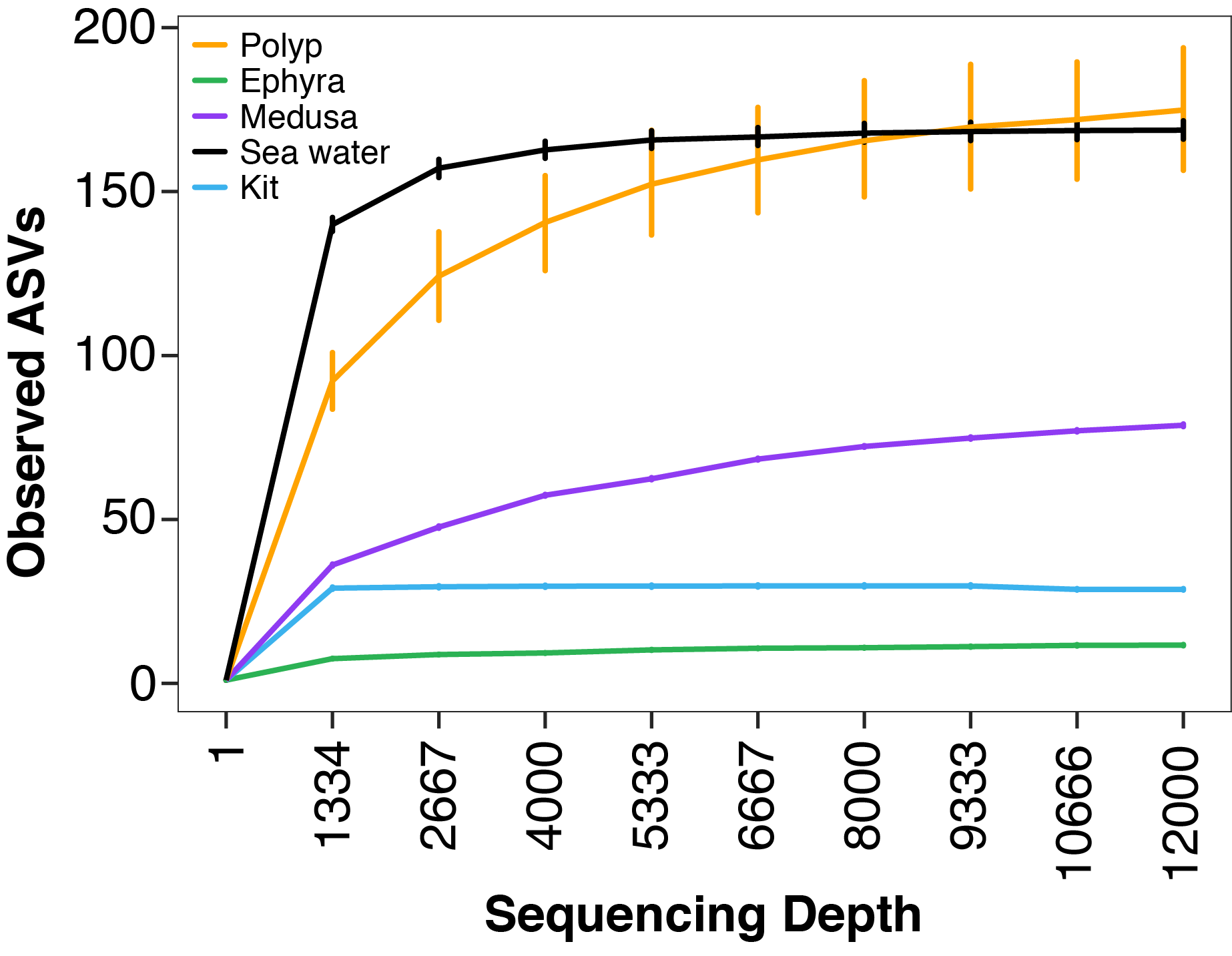


**S1 Figure**

**The alpha-rarefaction curve**

Samples are grouped by life stage. The number of ASVs are averaged by sample type. Reads were sub-sampled to a depth of 12,000, which is approximate to the lowest read depth of the jellyfish samples, ensuring all samples are represented in the plot. Error bars are bootstrapped confidence intervals calculated with the Seaborn data visualization software in Python.


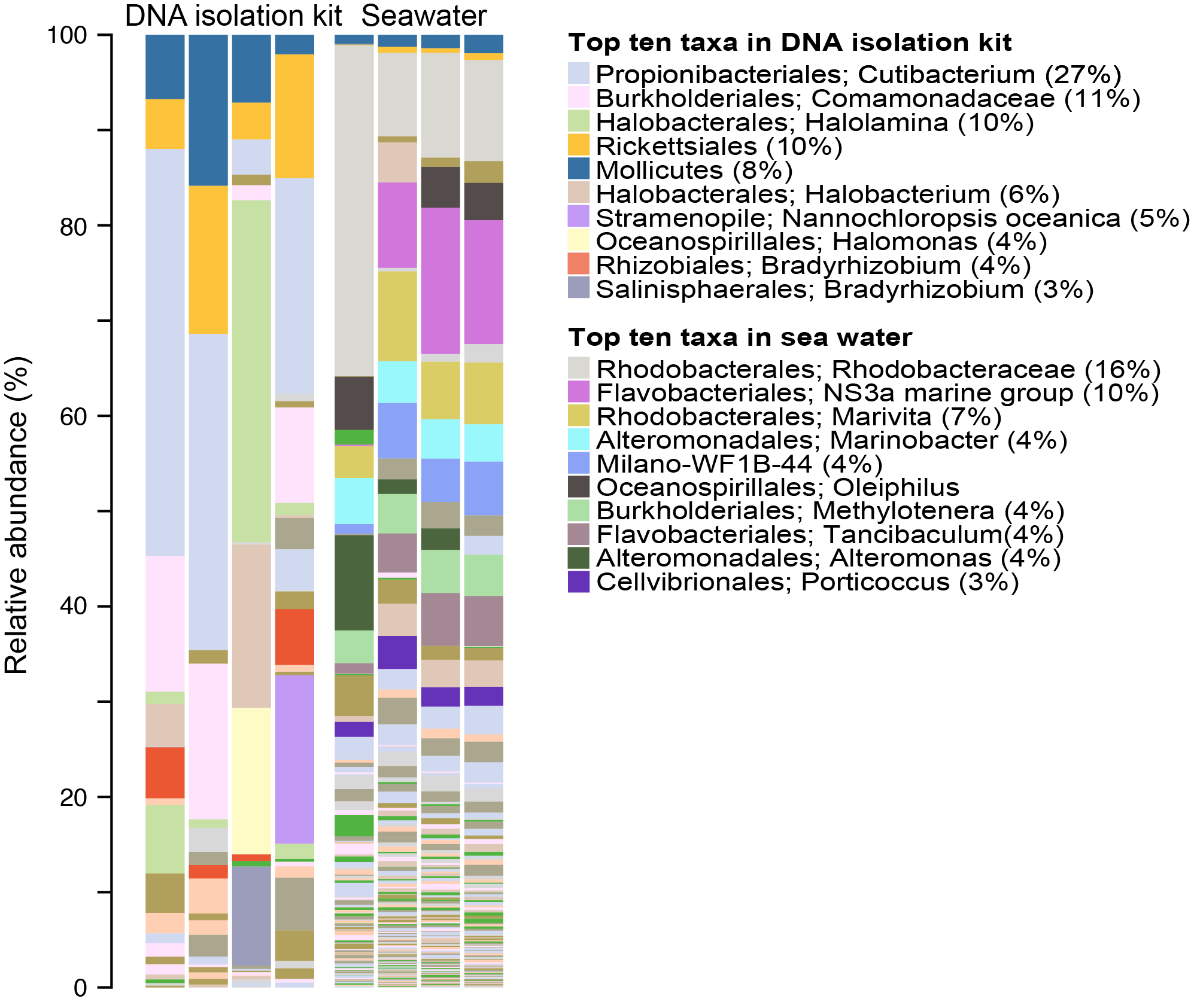


# S2 Figure

**Microbial composition of 16S amplicon sequencing control samples**

As controls for the amplicon sequencing experiments, we sequenced the DNA isolation kit and sea water. We recovered common contaminants (e.g., Cutibacaterium) as top taxa in the DNA isolation kit, and common taxa associated with sea water (Rhodobacteraceae, Marinobacter, Flavocateria).

We also found *Aurelia* Mollicutes and *Aurelia* Rickettsiales in the kit (total 18%) and sea water control (< 2%). These are likely due to external contamination, which are commonly observed in 16S amplicon sequencing experiments (see Davis et al. 2018) and can occur for investigators with frequent contact with animal subjects or in laboratories directly housing the animals. To further verify the true microbiome of *Aurelia*, we used the bacterial composition from the control samples to run the contamination analysis as described in the main text and verified that *Aurelia* Mollicutes and Rickettsiales are with high likelihood genuine members of the moon jellyfish microbiome.


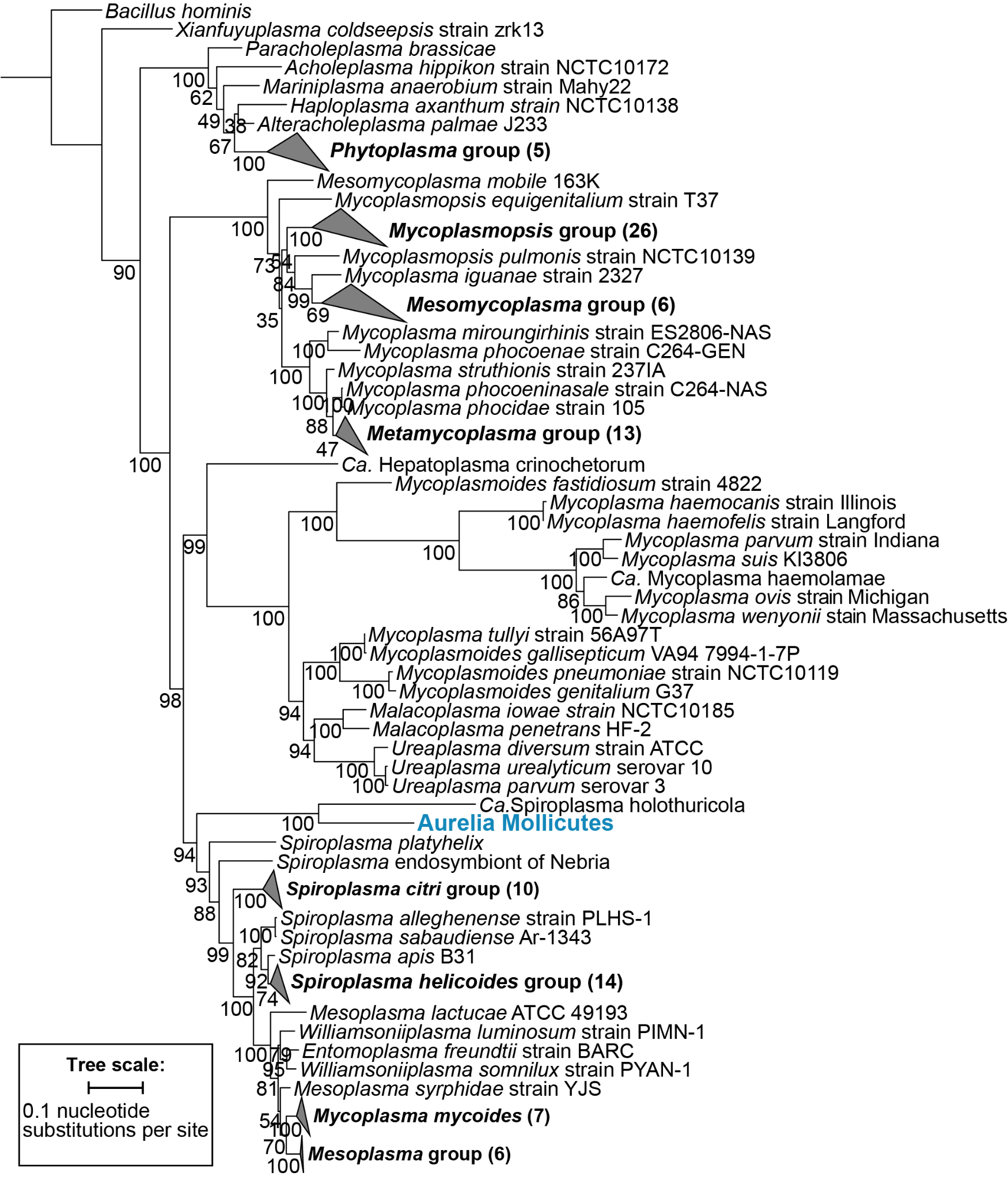


# S3 Figure

**Maximum-likelihood tree of Mollicutes 16S ribosomal RNA gene**

The phylogeny was reconstructed with IQ-TREE under the GTR+F+I+G4 model with ultrafast bootstrapping (UF; n=2000). UF bootstrapping support values are shown for each branch point. The number of species comprising collapsed branches are shown in parentheses.


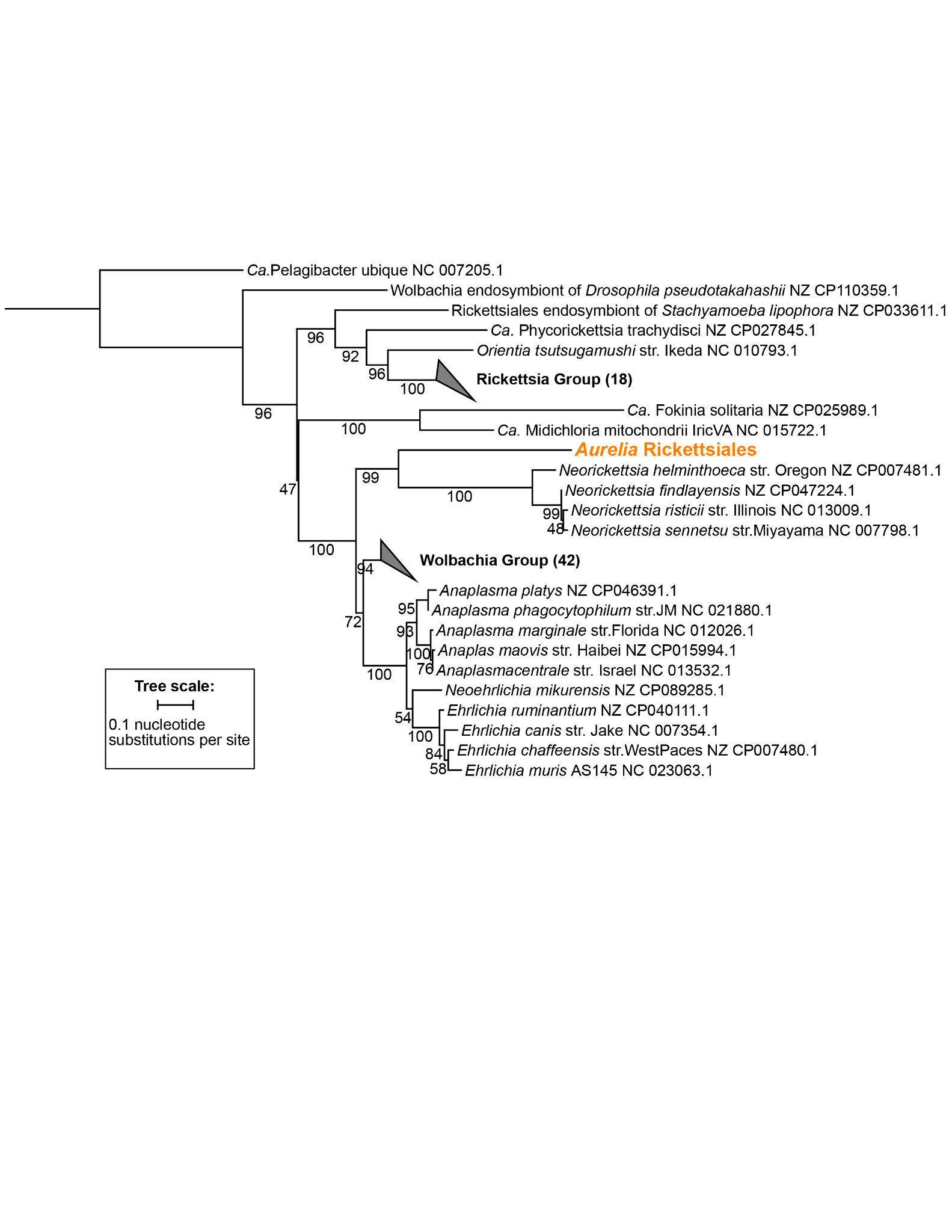


# S4 Figure

**Maximum-likelihood tree of Rickettsiales 16S ribosomal RNA gene**

The phylogeny was reconstructed with IQ-TREE under the GTR+F+I+G4 model with ultrafast bootstrapping (UF; n=2000). UF bootstrapping support values are shown for each branch point. The number of species comprising collapsed branches are shown in parentheses.
